# Factors associated with access to healthcare services for older adults with limited activities of daily living

**DOI:** 10.3389/fpubh.2022.921980

**Published:** 2022-10-06

**Authors:** Shumin Mai, Jingjing Cai, Lu Li

**Affiliations:** ^1^The Institute of Social and Family Medicine, School of Medicine, Zhejiang University, Hangzhou, China; ^2^Shulan International Medical College, Zhejiang Shuren University, Hangzhou, China

**Keywords:** access to healthcare services, activities of daily living, Andersen's healthcare utilization model, older adult, active aging

## Abstract

**Background:**

Limited studies focused on the situation and related factors of access to healthcare services for older adults with limited activities of daily living (ADL) in China. This study explores factors associated with access to healthcare services of them based on Andersen's healthcare utilization model (namely, need, predisposing, and enabling dimensions).

**Methods:**

A total of 3,980 participants aged 65 years and older adults with limited ADL from the latest wave (2018) of the Chinese Longitudinal Healthy Longevity Survey (CLHLS) were included. Binary logistic regression was used to explore the influencing factors.

**Results:**

Factors in enabling dimension were associated with access to healthcare services for older adults with limited ADL. Those who lived with better economic status (fair vs poor, OR = 2.98, *P* < 0.01; rich vs poor, OR = 7.23, *P* = 0.01), could afford daily life (yes vs no, OR = 2.33, *P* = 0.03), and lived in the eastern or central region of China (eastern vs western, OR = 2.91, *P* < 0.01; central vs western, OR = 2.40, *P* = 0.02) could access to healthcare services more easily. However, factors in predisposing dimension and need dimension showed no statistical significance. Meanwhile, inconvenience in the movement was the major barrier reported by some participants for not going to the hospital when they got sick.

**Conclusion:**

Access to healthcare services for older adults with ADL limitation was mainly related to the factors of economic status, affordability for daily life, and living regions in enabling dimension. Strategies focused on health insurance, healthcare system, barrier-free facilities, and social support were proposed to increase the access to healthcare services for participants, which could benefit their health.

## Introduction

The number of Chinese older adults over the age of 60 and 65 had reached 264 million, and 190 million, respectively, by 2020 (1). The former accounts for 18.70% of the total population, and the latter is 13.50% (1). It is estimated that the population who are 65 years or above would reach 329 million in 2050, accounting for 29% of the entire population in China (2). Because of physiological fragile, older adults may get sick more easily than young people or the general population (3). It was reported that among older adults aged 65 or older, 75% lived with health problems and about 54% had non-communicable diseases (NCDs) in 2017 (4). These indicated that older adults would have more demand for healthcare services than other populations. Access to healthcare services is crucial to maintaining physical function and quality of life for older adults. Access has not been defined or employed precisely (5, 6). To some research “access” refers to entry into or use of the healthcare system or characterizes factors influencing entry or use, while to others it is described as the degree of fit between the patient and the healthcare system and includes specific dimensions, such as availability, accessibility, accommodation, affordability, and acceptability (6). Thus, we conclude that access could be described as the degree or the difficulty of accessing health services for the population when they need them. Organizational, financial, or cultural restrictions may lead to inadequate access to healthcare services, thus, in turn, causing detrimental effects on social and individual levels (7). Relevant studies demonstrated that enough access to healthcare services could improve the odds of survival and healthy survival at old and very old ages (8). While inadequate access would increase the odds of physical disability, cognitive impairment, and mortality (9, 10). China has adopted related policies to increase the opportunity for older adults to get healthcare services in recent years (11). Take the example of family doctor services in China. It was first proposed by the government in 2016. Doctors make regular follow up to older adults, especially to those with disabilities or mobility problems, and provide them with health care services (12). However, limitations in related insurance policies, the shortage of family doctors, and other factors contributed to unsatisfied healthcare services demand for older adults (12). Thus, the policy still needed to be improved. It is reported that the number of older adults with limitations in activities of daily living (ADL) would increase from 8.4 million in 2010 to 37 million in 2050 in China (4). This vulnerable group may experience depressive symptoms, worse quality of life, poor subjective wellbeing, functional impairment, or suicide (13–15). Having adequate healthcare services is one of the important ways to maintain their health.

In China, many studies explored the access to healthcare services for migrant population (16–18), rural–urban residences (10), participants with different social demographics (9), and patients' caregivers (19). Compared with the research participants mentioned above, studies focused on older adults with limited ADL were relatively limited. Relevant research on older adults with disability conducted in China included small sample size and cover a few investigation sites (20). Meanwhile, many studies about influencing or related factors about access to healthcare services for older adults carried out in China usually lacked systematic theory research frameworks (18, 21, 22). Therefore, this study aims to (1) picture the situation of access to healthcare services for older adults with limited ADL based on the CLHLS database which covered a large sample and research areas in China, and (2) explore the related factors associated with access to healthcare services for older adults with limited ADL based on Andersen's healthcare utilization model.

## Methods

### Patient and public involvement

Data were extracted from the Chinese Longitudinal Healthy Longevity Survey (CLHLS), which monitors the health status and its determinants of older adults in China (23). The CLHLS collected eight waves of data in 1998, 2000, 2002, 2005, 2008, 2011, 2014, and 2018. It consists of rich information, such as sociodemographic characteristics, health status, daily activities living of older adults, and so on. Participants in the CLHLS were recruited by a targeted random sampling process, by which investigators firstly recruit an eligible centenarian interviewee in sampled city/county, and then matched a nonagenarian, octogenarian, and three elders aged 65–79 nearby in the same street, village, or town (24, 25). Then, participants were interviewed face-to-face by investigators. For those who are not able to answer the questions, a close family member or another knowledgeable proxy (significant other) was invited to provide answers (24). The CLHLS included a large sample of the oldest-old (>=80 years old). For example, it recruited 8,805 older persons aged 80 to 105 in the 1998 wave (24). Meanwhile, those older adults who were interviewed but subsequently died before the next wave would be replaced by new interviewees of the same sex and age (or within the same 5-year age group) (24). Currently, there are many studies on the oldest-old based on CLHLS (26–28). And the reliability of age reporting based on CLHLS was confirmed (23). More details of CLHLS have been described elsewhere (25), and could be obtained from its website (http://opendata.pku.edu.cn/dataverse/CHADS).

**Figure 1:**
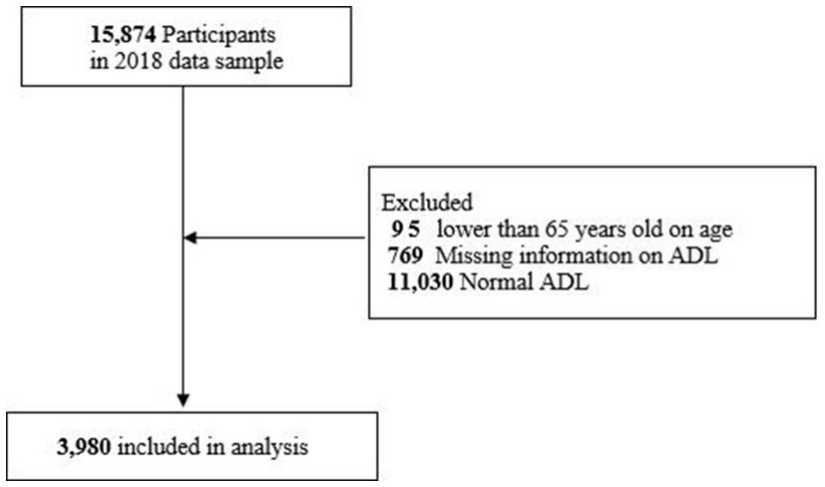
Flowchart of participants.

In this study, we used the latest wave (2018) of the CLHLS. Participants aged 65 years and above in the database were included in our study. Those aged 64 and below would be excluded. Because we need to analyze the related factors of access to healthcare services among older adults with limited ADL. Those who had missing information on ADL and older adults with normal ADL would be excluded. The final sample size was 3,980 (Figure 1).

### Ascertainment of limited activities of daily living (ADL)

Activities of daily living (ADL) were measured by six items: bathing, dressing, toileting, indoor transferring, continence, and feeding. Older adults were given 1 score (complete dependence on others), 2 scores (partial independence), or 3 scores (complete independence) when answering the independence ability to complete the above actions. The total score ranges from 6 to 18, and a higher score indicates better daily living ability. If the older adult needed assistance in any one of the six items, namely the total score lower than 18, he/she is defined to have ADL limitation. This measurement was used and its accessibility was confirmed by previous studies (29, 30).

### Ascertainment of access to healthcare services

In this study, we measured access to healthcare services by using a self-report single question: “Could you get adequate medical services when it is necessary?”. Respondents were asked to choose “yes” or “no” according to their perception. This question was used in each cohort wave of CLHLS, and self-reported could reflect the situation and evaluation of access to healthcare services perceived by older adults. It could be seen as a complement to the objective situation of access to healthcare services and provide a reference to guide the development of related policies. Meanwhile, the measurement approach has been widely used in previous CLHLS-based studies and the measurement quality has been confirmed (2, 8–10, 31).

### Explanatory variables

The explanatory variables included in this study were categorized according to the Andersen healthcare utilization model that includes need, predisposing, and enabling dimensions (32). It was regarded as a useful model to analyze healthcare utilization and has been widely used to explore people's health behavior and influencing factors (33, 34). Need indicates whether and what healthcare services are needed by an individual (32). Predisposing determines the inclination of an individual to seek healthcare services and enabling refers to activating or impeding the realization of healthcare-seeking behaviors of those in need (32). We used the investigation tool constructed by previous scholars to measure the above three dimensions based on the CLHLS database (2). Older adults' need for healthcare services was measured by two items. They were asked to rate their overall health on a five-point Likert scale ranging from “very bad” to “very good.” They were also asked to confirm whether they had hypertension, diabetes, and heart disease, which are the three most common chronic conditions in China. The former item could be seen as a subjective need and the latter as an objective need for healthcare services. In terms of predisposing, it was measured by demographic characteristics (age, gender, schooling, and marital status) and living arrangements (living alone or not) of the respondents. The dimension of enabling was measured by financial affordability (including self-rated financial status on a five-point Likert scale ranging from “very poor” to “very rich,” self-rated affordability for daily life that the answer was classified as “yes” or “no,” and self-reported the total number of out-of-pocket payment for medical care last year), employment status (retired or employed), the amount of health insurance coverage, residence (rural or city/town), and geographic location (western, central, eastern). The details of the items are shown in Supplementary Table 1.

### Covariate

The total score of ADL could reflect participants' health status. Older adults with more numbers of limited activities would get a lower score on ADL evaluation, which shows that older adults depend more on others in daily life and indicates they have poor health. Thus, the total score of ADL could be the potential influencing factor when analyzing the relationship between explanatory variables and access to healthcare services. Therefore, participants' ADL total score was regarded as a covariate in our study.

### Statistical analysis

SPSS version 20.0 was used to clean the data. Multiple imputation (MI) and binary logistic regression were used to replace missing data and explore the relationship between explanatory variables and access to healthcare services, respectively, with RStudio software. In this study, all the variables with missing data would be replaced by multiple imputation by chained equations (MICE) function in RStudio (35). MI is an advanced missing data handling method, which could replace continuous or categorical variables with missing data. In the process of MI, each missing value would be replaced by several different values and then several different completed datasets are generated. The imputed datasets are each analyzed and the study results are then pooled into the final study result. MICE was proposed by Buuren (36). In the MICE algorithm, a chain of regression equations is used to obtain imputations, which means that variables with missing data are imputed one by one.

## Results

### Social-demographic characteristics

The original characteristics of the participants without multiple imputation are shown in Supplementary Table 1. Missing data was filled with multiple imputation for variables such as self-rated health, chronic disease, years of schooling, marriage status, living arrangement, economic status, affordability for daily expenses, out-of-pocket payment for medical care, employment status, insurance, and access to healthcare services would be replaced. After replacing the missing data, most participants reported they had fair or good health. There was 67.21% of the participants who had chronic diseases. Most participants were between the ages of 80 and 99 years. Proportions of women participants were higher than men (67.21 vs 32.79%). The average years of schooling for the participants were low, namely 2.02 years. The proportion of the widowed participants was 82.29%. Most participants reported living with others, including living with family members or living in an institution. While those living alone accounted for a small proportion. The economic status of many participants was at a fair level. There was 83.72% of the participants could afford daily life. The average cost of out-of-pocket payment for medical care of the participants was 3780.81 yuan. And the standard deviation was large, which indicates the great difference in self-economic burden among the participants. More than half of older adults reported having retired. Most participants had one insurance. Those living in the city/town accounted for 60.10%, and the majority of participants were living in the eastern region of China. There were a few participants who could not access healthcare services when necessary (Table 1). The original characteristics of participants without multiple imputation are shown in Supplementary Table 1.

**Table 1:** Characteristics of participants after multiple amputation (*N* = 3,980).

		**Frequency**	**Percentage (%)/**
		**(*n*)**	**Mean ±SD**
**Need dimension**			
	**Self-rated health**		
	Very bad	129	3.30
	Bad	807	20.28
	Fair	1,497	37.61
	Good	1,203	30.23
	Very good	344	8.64
	**Chronic disease**		
	No	1,305	32.79
	Yes	2,675	67.21
**Predisposing dimension**			
	**Age**		
	65–79	258	6.48
	80–99	1,980	49.75
	>=100	1,742	43.77
	**Gender**		
	Male	1,305	32.79
	Female	2,675	67.21
	**Years of schooling (continuous measurement)**	3,980	2.02 ± 3.79
	**Marriage status**		
	Married	630	15.83
	Separated/divorced	52	1.31
	Widowed	3,275	82.29
	Never married	23	0.58
	**Living arrangement**		
	Alone	334	8.39
	Living with others	3,546	89.10
**Enabling dimension**			
	**Economic status**		
	Very poor	86	2.16
	Poor	455	11.43
	Fair	2,728	68.54
	Rich	598	15.03
	Very rich	113	2.84
	**Affordability for daily life**		
	No	648	16.28
	Yes	3,332	83.72
	**Out-of-pocket payment for medical care (continuous measurement)**	3,980	3780.81 ± 11575.83
	**Employment**		
	Retired	2,109	52.99
	Employed	1,871	47.01
	**Insurance**		
	No	374	9.40
	One	1,955	49.12
	Two or above	1,651	41.48
	**Residence**		
	Rural	1,588	39.90
	City/town	2,392	60.10
	**Region** ^a^		
	Western	786	19.75
	Central	1,002	25.18
	Eastern	2,192	55.08
**Access to health services**			
	No	192	4.82
	Yes	3,788	95.18
**Limited ADL total score (continuous measurement)**		3,980	13.13 ± 3.44

The percentage of missing information from the original data ranged from 0.80–69.95, more detail was shown in Supplementary Table 1.

^*a*^Areas were usually divided according to their geographical and economic status in China. The investigation areas of CLHLS covers 23 of 31 provinces in China mainland. Among them, Guangxi Zhuang Autonomous Region, Chongqing, Sichuan Province, and Shaanxi Province were classified as western region; Shanxi Province, Jilin Province, Heilongjiang Province, Anhui Province, Jiangxi Province, Henan Province, Hubei Province and Hunan Province as central region; and Beijing, Tianjin, Hebei Province, Liaoning Province, Shanghai, Jiangsu Province, Zhejiang Province, Fujian Province, Shandong Province, Guangdong Province and Hainan Province as eastern region. Eastern region usually ranks first in average GDP, followed by central region, and western region (37, 38). CLHLS would expand its investigation areas in the future study.

### Related factors of access to healthcare services among older adults with limited ADL

To reduce interaction and collinearity between different variables, we reclassified the variables and combined those categories with a few samples (Supplementary Table 2). For example, in self-rated health “very bad” and “bad,” “very good” and “good” of the original data were reclassified as “bad,” and “good,” respectively. After multiple imputation, logistic regression analysis was used to explore the relationship between the explanatory variables and access to healthcare services. After adjusting the covariates, the analysis results demonstrated that factors in the dimensions of need and predisposing did not have statistical significance. In enabling dimension, older adults with better economic status (fair vs poor, OR = 2.98, *P* < 0.01; rich vs poor, OR = 7.23, *P* = 0.05), could afford daily life (yes vs no, OR = 2.33, *P* = 0.03), and living in central or eastern China (central vs western, OR = 2.40, *P* = 0.02; eastern vs western, OR = 2.91, *P* < 0.01) could access to healthcare services easily compared with those with poor economic status, could not afford for daily life, and living in western China. The detailed information are shown in Table 2.

**Table 2:** Related factors of access to healthcare services among participants.

**Explanatory variables (reference group)**		**B**	***P***	**OR**	**95%*CI***
**Need dimension**					
	**Self-rated health (Bad)**				
	Fair	2.09	0.47	1.76	1.08–2.89
	Good	1.05	1.40	1.33	0.72–2.46
	Chronic disease (No)	1.07	0.40	1.34	0.94–1.91
**Predisposing dimension**					
	**Age**				
	80–99	0.84	2.06	1.26	0.58–2.72
	>=100	0.52	2.72	1.15	0.50–2.62
	**Gender (Male)**	−0.61	1.64	0.85	0.55–1.30
	**Years of schooling (continuous measurement)**	0.35	3.08	1.01	0.94–1.08
	**Marriage status (Married)**	−2.06	1.08	0.57	0.31–1.07
**Enabling dimension**					
	**Living arrangement (Alone)**	1.21	0.78	1.39	0.83–2.33
	**Economic status (Poor)**				
	Fair	1.09	<0.01	2.98	2.03–4.37
	Rich	1.98	0.01	7.23	3.12–16.72
	**Affordability for daily life (No)**	3.11	0.03	2.33	1.56–3.47
	**Out-of-pocket payment for medical care (continuous measurement)**	0.02	1.67	1.00	0.99999–1.00003
	**Employment status (Retired)**	−1.19	2.69	0.92	0.56–1.50
	**Insurance (No)**				
	> =1	0.67	1.73	1.20	0.73–1.97
	**Residence (Rural)**	0.72	1.02	1.22	0.85–1.73
	**Region (Western)**				
	Central	3.22	0.02	2.40	1.59–3.61
	Eastern	1.07	<0.01	2.91	2.02–4.18
**Covariate variable**	ADL total score	1.12	0.06	1.09	1.03–1.14

95%CI, 95% confidence interval.

### Barriers to using health services

Participants also reported their reasons for not going to the hospital when they get sick. Among 253 participants who answered this question. The top three reasons were inconvenience in movement (43.48%, 110/253), insufficient money (20.16%, 51/253), and unwillingness to go (16.60%, 42/253).

## Discussion

This study assessed the factors associated with access to healthcare services for older adults with limited ADL based on Andersen's healthcare utilization model. We found that the access to healthcare services of older adults with limited ADL was related to factors in enabling dimension. In contrast, factors in the dimensions of need and predisposing did not have statistical significance. Meanwhile, some participants did not go to the hospital when they got sick. Inconvenience in the movement was the first reason, followed by insufficient money and unwillingness to go.

In terms of the factors associated with access to healthcare services for older adults with limited ADL. After controlling the confounding factors, the analysis result showed that the dimension of need did not have statistical significance. Self-rated health and chronic disease could be seen as subjective and objective needs of healthcare services, respectively (2). Usually, need is the precondition of health services utilization. However, related studies showed that people who were not in need may still be able to access healthcare when they need it (39). Furthermore, the concepts of need and demand are different. In health economics, demand is the amount of a good or service that consumers are willing and able to buy at varying prices (40). While need relates to the number of goods or services which should be consumed based on professional value judgments, without taking the ability to pay into consideration (40). Thus, it did not have statistical significance. In terms of health, WHO defined it as a state of complete physical, mental, and social wellbeing and not merely the absence of disease and infirmity (41). In this study, many older adults reported they had fair or good health conditions although they had limited ADL or physical limitations. Probably because older adults had a positive attitude toward life, namely good subject health. The previous related research based on CLHLS showed that long-lived individuals or oldest-olds had positive subjective wellbeing or life satisfaction despite constraints in objective life conditions (23). Meanwhile, Confucianism is an important traditional culture that influences the attitudes and behavior of Chinese people, and it promotes optimism when facing difficulties (42). However, the potential reasons deserved further research in future as CLHLS provided limited information about this. Furthermore, in this study, more than 90% of older adults reported they lived with others. They could get help from family members or caregivers when they were in difficulties. A positive attitude toward life or a completed family support network may contribute to better mental health for them. Thus, it may result in no statistical significance.

Access to healthcare services for older adults with limited ADL was mainly related to the factors in enabling dimension. Older adults who could afford daily life could access health services more easily than those who could not. Participants with better economic status reported that they could get adequate medical services when it is necessary compared with older adults with poor economic status. Older adults living in eastern or central regions could gain health services more easily than those living in the western region. The results above are consistent with relevant studies (20, 22, 43). When talking about economic status and affordability for daily life, these two factors indicated that financial capacity plays an important role in access to healthcare services for older adults with limited ADL. Compared with young people, older adults suffer from different kinds of diseases and impairments and would spend more money on health services (44). Thus, enough financial capacity is one of the basic conditions that ensure they gain sufficient healthcare services. In terms of living regions, the advanced and excellent healthcare resources are mainly distributed in the eastern region of China, followed by the central region, while the western region is relatively insufficient (45, 46). The unbalanced healthcare resources distribution hinders older adults who are living in remote areas from getting immediate or enough healthcare services (37). The analysis result revealed that other enabling factors, such as out-of-pocket payment, employment status, insurance, and residence, did not have statistical significance. Probably because of the interaction effect or similar distribution of different variables. Taking insurance, for example, most participants had at least one insurance. Over the past few decades, China has made important progress in achieving full insurance coverage across and within regions (37). In 2018, health insurance coverage in China was 96.8% of the whole population (47). It helps to reduce some economic burdens for patients, and the high insurance coverage indicates that most Chinese people had basic healthcare protection. Therefore, the analysis result did not have statistical significance.

Factors that consisted of predisposing dimensions did not associate with the access to healthcare services of the participants, which was different from the relevant studies (9, 10). Probably because compared with other factors, like mobility or physical ability, predisposing had a smaller impact on getting healthcare services for older adults with limited ADL. Furthermore, predisposing dimension includes many social demographic characteristics, and they may have potential interaction with each other or older adults with limited ADL had similar social demographic characteristics distribution. Therefore, it did not have statistical significance. Meanwhile, China has conducted healthcare reform in recent years. Many healthcare policies were conducted to let people, especially those living in poverty or remote areas, enjoy different kinds of healthcare services near their residences (11). For example, integrated healthcare system, telemedicine, and family doctor services have been promoted greatly in recent years. The integrated healthcare system refers to the tertiary hospitals as the core, combined with the secondary hospitals in the region and the community health service centers (11). By optimizing and integrating the medical resources, the community or remote areas residents can enjoy homogenized services, thus creating a reasonable and orderly medical treatment model (11). These policies could realize geographic and service accessibility and thus social demographic characteristics may have a minor influence over other factors. However, these policies themselves and their implementation were confronted with many problems, which means more efforts are needed. For example, the application of telemedicine is relatively limited in intensive care and nursing because of the immature technology, lacking legislation on charging standards, and responsibility distinction (46). And it gains low acceptance by patients especially older adults who are unfamiliar with information technology (48). With the increasing population and older adults, the total amount of family doctors was insufficient (49). In terms of the development of an integrated healthcare system, it was limited by the inadequate technical level of secondary or primary services centers (11), the fragmented governance, and incoordination of healthcare resources in China (50, 51). Furthermore, these policies could not change the unbalanced distribution of excellent or high-quality medical resources between different regions or spatial disparities in a short time, many provinces with low GDP levels or low urbanization, such as provinces in the western region, those resources were in shortage relatively (45). In addition, these policies are focused on the general population while people with limited ADL deserved further attention. Meanwhile, relevant studies (52, 53) indicated that policies usually had delayed or lag effects. Policies about access to healthcare services usually include comprehensive and complicated issues. Therefore, it needs more time to bring these policies into effect. Thus, this study figured out that access to healthcare services still varies in different regions.

Although participants had a high rate of access to healthcare services, some older adults did not go to hospitals when they got sick. This study found that the primary reason was an inconvenience in movement. Hospitals or medical institutions had professional medical staff, equipment, drugs, and other medical resources, which could provide comprehensive and professional healthcare services for patients. Adequate access to healthcare is beneficial to health by facilitating timely and quality medical care to screen for and treat diseases in the early stage, postponing declines in physical function with illness, restoring immune function, and ultimately prolonging survival (10). However, older adults with ADL limitations or mobility disabilities would be impeded getting healthcare services such as going to the hospital immediately, especially those who lack family members or caregivers' help (9). Related studies indicated that transport problems hinder disabled people from getting healthcare services (43). In China, there are still some difficulties for disabled people going out currently. Although many public transportations have installed barrier-free facilities. Too much traffic during regular work days, low service awareness of the drivers, and many time and human resources needed to switch on these facilities contribute to low usage of the facilities for disabled people (54). It results in difficulty of geographic accessibility for people with disability to access healthcare services. Therefore, the phenomenon or problem mentioned above may become the potential reason why inconvenience in movement is the major reason that participants did not go to hospitals when they got sick.

The analysis results about related factors of access to healthcare services based on Andersen's healthcare utilization model and self-reported reasons for not going to hospitals demonstrated that relevant policies should be considered to improve the accessibility to healthcare services for older adults with limited ADL. In terms of financial factors, both health and endowment insurances should be improved to reduce older adults' economic burden and increase their willingness to utilize healthcare services instead of bearing the pain of disease. Currently, although China has made large insurance coverage across and within regions, it is still at the basic level. The depth and scope of the insurance policy, such as health insurance reimbursement rates and coverage of different kinds of diseases needed to be increased or expanded (22, 37). When talking about the distribution of healthcare resources. Policies like integrated healthcare system, telemedicine, and family doctor services mentioned above should be further promoted and implemented so that older adults in remote or poorer areas could gain healthcare services immediately. Inconvenience in mobility is the main reason that older adults with limited ADL reported why they did not go to hospitals when they were in need. First, enough family doctors could provide medical services by going to older adults' homes immediately when they are in need. Meanwhile, related studies recommended that all efforts be expended to extend appropriate rehabilitation services, including home-based care and appliances to those identified as having a disability, particularly to those older than 65 years (43, 55). And the services need to be affordable and accessible in terms of suitable transport so that older adults with low income and mobility problems could enjoy these services (43, 55). Second, barrier-free facilities should be installed widely in public areas. Government or community managers could provide regular public transportations, especially for older adults with limited ADL to help them go to hospitals nearby when they need it (56). In addition, relevant studies showed that family or social support could help older adults, especially those with limited ADL, access healthcare services more easily (8, 18). Therefore, family members or caregivers should take care of older adults with mobility limitations, and pay more attention to their health. They should accompany older adults to accept healthcare services as soon as possible when older adults are in need. For community managers or governments, family doctor services deserved more investment. Doctors should visit older adults regularly, especially those having inconvenienced in movement.

Our study analyzed the current situation of access to healthcare services for older adults with limited ADL based on a national representative database in China, CLHLS. This provides us with a comprehensive insight into the healthcare services usage of older adults with limited ADL across the country. In addition, we adopted Andersen's healthcare utilization model to systematically explore related factors of access to healthcare services among older adults with limited ADL. Meanwhile, relevant strategies were proposed to increase financial and geographical accessibility to healthcare services based on a complete look into related factors. For example, we suggested policy priority should be put into the appropriate increase of health insurance reimbursement rate, even distribution of healthcare resources across regions, and growing investment in barrier-free facilities. On top of that, we call on more involvement of family members in elderly care. However, there were some limitations in our study. First, access to healthcare services was measured by one item in this study due to limited related information from the CLHLS database. It may lack information about objective service utilization. However, self-reported access to healthcare services could indicate if the objective service utilization could satisfy participants' needs and show if the supply of the healthcare services could help older adults. It could be one of the most important information to guide the formation of related policies. Furthermore, there are many variables in CLHLS, and investigating access to healthcare services was not the only goal of it. In future study, we could try to include representative items and develop scales to measure related variables systematically. Second, followed-up studies or cohort studies should be conducted to fully analyze in future as this research was confirmed based on cross-sectional analysis. Last but not the least, in future study, other related variables or influencing factors, such as social support, social network, patients' medical expectation, patients' health-seeking behavior or preference, and patients' health literacy, could be included to fully realize the situation and related factors of access to healthcare services of older adults with limited ADL.

## Conclusion

In this study, we found that access to healthcare services for older adults with ADL limitations was mainly related to the factors of economic status, affordability for daily life, and living regions in enabling dimension based on Andersen's healthcare utilization model. While factors in the dimensions of need and predisposing did not have statistical significance. Meanwhile, inconvenience in the movement was the major barrier that some participants reported why they did not go to the hospital when they got sick. Strategies related to health insurance, healthcare system, barrier-free facilities, and social support were proposed to increase the access to healthcare services for older adults with limited ADL, which could benefit their health.

## Data availability statement

The dataset could be applied on the CLHLS website: https://opendata.pku.edu.cn/dataverse/CHADS.

## Ethics statement

This study is a secondary analysis of the data from the CLHLS. The CLHLS was approved by the Ethics Committee of Peking University (IRB00001052-13074). Written informed consent was obtained from all participants for their participation in CLHLS study.

## Author contributions

SM contributed to the design of the study, sorting and analysis of data, and wrote the manuscript. JC participated in discussing the idea and helping to revise manuscript. LL helped to modify articles and supervised the whole study. All authors have read and approve the final manuscript.

## Funding

This study was funded by the National Social Science Fund of China in 2021 (No. 21BGL235).

## Conflict of interest

The authors declare that the research was conducted in the absence of any commercial or financial relationships that could be construed as a potential conflict of interest.

## Publisher's note

All claims expressed in this article are solely those of the authors and do not necessarily represent those of their affiliated organizations, or those of the publisher, the editors and the reviewers. Any product that may be evaluated in this article, or claim that may be made by its manufacturer, is not guaranteed or endorsed by the publisher.
