# Supplementary material for: Factors associated with access to healthcare services for older adults with limited activities of daily living
Source: Front Public Health. 2022 Oct 6;10:921980. doi: 10.3389/fpubh.2022.921980 (PMC9583939; doi:10.3389/fpubh.2022.921980)
Supplement: Supplementary file 1 [file Data_Sheet_1.docx]

**Supplementary material**

**1. Original characteristics of participants**

The original data had a similar distribution to those data after multiple imputation. For example, most participants reported that they had fair or good health, living with others, had fair economic status, could afford daily expenses, retired, participated in one insurance, and could access healthcare services. The majority of the participants with window marriage status. Meanwhile, the average years of schooling for the participants was low, namely 2.05 years.

**Table1 Original characteristics of the participants without multiple imputation (N = 3,980)**

|  |  | Frequency (n) | Percentage(%)/Mean ± SD |
| --- | --- | --- | --- |
| **Need dimension** |  |  |  |
|  | **Self-rated health** |  |  |
|  | Very bad | 97 | 2.44 |
|  | Bad | 617 | 15.50 |
|  | Fair | 1,13 | 28.57 |
|  | Good | 886 | 22.26 |
|  | Very good | 275 | 6.91 |
|  | Missing | 968 | 24.32 |
|  | **Chronic disease** |  |  |
|  | No | 1,869 | 46.96 |
|  | Yes | 1,507 | 37.86 |
|  | Missing | 604 | 15.18 |
| **Predisposing dimension** |  |  |  |
|  | **Age** |  |  |
|  | 65-79 | 258 | 6.48 |
|  | 80-99 | 1,980 | 49.75 |
|  | >=100 | 1,742 | 43.77 |
|  | **Gender** |  |  |
|  | Male | 1,305 | 32.79 |
|  | Female | 2,675 | 67.21 |
|  | **Years of schooling**  (continuous measurement) | 3,980 | 2.05$\pm$3.85 |
|  | Missing | 456 | 11.46 |
|  | **Marriage status** |  |  |
|  | Married | 622 | 15.63 |
|  | Separated/divorced | 50 | 1.26 |
|  | Widowed | 3,246 | 81.56 |
|  | Never married | 23 | 0.58 |
|  | Missing | 39 | 0.98 |
|  | **Living arrangement** |  |  |
|  | Alone | 330 | 8.29 |
|  | Living with others | 3,613 | 90.78 |
|  | Missing | 37 | 0.93 |
| **Enabling dimension** |  |  |  |
|  | **Economic status** |  |  |
|  | Very poor | 83 | 2.09 |
|  | Poor | 447 | 11.23 |
|  | Fair | 2,675 | 67.21 |
|  | Rich | 590 | 14.82 |
|  | Very rich | 111 | 2.79 |
|  | Missing | 74 | 1.86 |
|  | **Affordability for daily expenses** |  |  |
|  | No | 640 | 16.08 |
|  | Yes | 3,308 | 83.12 |
|  | Missing | 32 | 0.80 |
|  | **Out-of-pocket payment for medical care**  (continuous measurement) | 3,980 | 3,785.93$\pm$11,561.60 |
|  | Missing | 474 | 11.91 |
|  | **Employment status** |  |  |
|  | Retired | 919 | 23.09 |
|  | Unretired | 277 | 6.96 |
|  | Missing | 2,784 | 69.95 |
|  | **Insurance** |  |  |
|  | No | 326 | 8.19 |
|  | One | 1,749 | 43.94 |
|  | Two or above | 1,495 | 37.56 |
|  | Missing | 410 | 10.30 |
|  | **Residence** |  |  |
|  | Rural | 1,588 | 39.90 |
|  | City/town | 2,392 | 60.10 |
|  | **Region ^a^** |  |  |
|  | Western | 786 | 19.75 |
|  | Central | 1,002 | 25.18 |
|  | Eastern | 2,192 | 55.08 |
| **Access to health services** |  |  |  |
|  | No | 190 | 4.77 |
|  | Yes | 3,735 | 93.84 |
|  | Missing | 55 | 1.38 |
| **Limited ADL total score**  (continuous measurement) |  | 3,980 | 13.13$\pm$3.44 |

Note: ^a^ Areas were usually divided according to their geographical and economic status in China. The investigation areas of CLHLS cover 23 of 31 provinces in China mainland. Among them, Guangxi Zhuang Autonomous Region, Chongqing, Sichuan Province, and Shaanxi Province were classified as western region; Shanxi Province, Jilin Province, Heilongjiang Province, Anhui Province, Jiangxi Province, Henan Province, Hubei Province and Hunan Province as central region; and Beijing, Tianjin, Hebei Province, Liaoning Province, Shanghai, Jiangsu Province, Zhejiang Province, Fujian Province, Shandong Province, Guangdong Province and Hainan Province as eastern region. Eastern region usually ranks first in average GDP, followed by central region, and western region. (References: [1] Meng Q,Xu L, Zhang Y, Qian J, Cai M, Xin Y,et al.Trends in access to health services and financial protection in China between 2003 and 2011: a cross-sectional study, Lancet,2012,379(9818):805-814. [2] Statistical Communique of the People’s Republic of China on the 2019 National Economic and Social Development released by National Bureau of Statistics. Available on http://www.stats.gov.cn/xxgk/sjfb/tjgb2020/202006/t20200617_1768655.html.) CLHLS would expand its investigation areas in the future study.

**2. Table 2 Reclassification of variables after multiple imputation(N=3,980)**

|  |  | Frequency (n) | Percentage(%)/Mean ± SD |
| --- | --- | --- | --- |
| **Need dimension** |  |  |  |
|  | **Self-rated health** |  |  |
|  | Bad | 1,026 | 25.78 |
|  | Fair | 1,503 | 37.76 |
|  | Good | 1,451 | 36.46 |
|  | **Chronic disease** |  |  |
|  | No | 2,205 | 55.40 |
|  | Yes | 1,775 | 44.60 |
| **Predisposing dimension** |  |  |  |
|  | **Age** |  |  |
|  | 65-79 | 258 | 6.48 |
|  | 80-99 | 1,980 | 49.75 |
|  | >=100 | 1,742 | 43.77 |
|  | **Gender** |  |  |
|  | Male | 1,305 | 32.79 |
|  | Female | 2,675 | 67.21 |
|  | **Years of schooling**  (continuous measurement) | 3,980 | 2.03$\pm$3.84 |
|  | **Marriage status** |  |  |
|  | Married | 630 | 15.83 |
|  | Separated/divorced/widowed/never married | 3,350 | 84.17 |
|  | **Living arrangement** |  |  |
|  | Alone | 333 | 8.37 |
|  | Living with others | 3,647 | 91.63 |
| **Enabling dimension** |  |  |  |
|  | **Economic status** |  |  |
|  | Poor | 542 | 13.62 |
|  | Fair | 2,724 | 68.44 |
|  | Rich | 714 | 17.94 |
|  | **Affordability for daily expenses** |  |  |
|  | No | 649 | 16.31 |
|  | Yes | 3,331 | 83.69 |
|  | **Out-of-pocket payment for medical care**  (continuous measurement) | 3,980 | 3,748.39$\pm$11,355.14 |
|  | **Employment status** |  |  |
|  | Retired | 2,235 | 56.16 |
|  | Unretired | 1,745 | 43.84 |
|  | **Insurance** |  |  |
|  | No | 370 | 9.30 |
|  | >= 1 | 3,610 | 90.70 |
|  | **Residence** |  |  |
|  | Rural | 1,588 | 39.90 |
|  | City/town | 2,392 | 60.10 |
|  | **Region** |  |  |
|  | Western | 786 | 19.75 |
|  | Central | 1,002 | 25.18 |
|  | Eastern | 2,192 | 55.08 |
| **Access to health services** |  |  |  |
|  | No | 193 | 4.85 |
|  | Yes | 3,787 | 95.15 |
| **Limited ADL total score**  (continuous measurement) |  | 3,980 | 13.13$\pm$3.44 |

Note: variables of self-rated health, marriage status, and economic status were reclassified. For example, in self-rated health “very bad” and “bad”, “very good” and “good” of the original data were reclassified as “bad”, “good” respectively; in economic status “very poor” and “poor”, “very rich” and “rich” of the original data were reclassified as “poor”, “rich” respectively.
